# In situ synthesis of Bi_2_S_3_ sensitized WO_3_ nanoplate arrays with less interfacial defects and enhanced photoelectrochemical performance

**DOI:** 10.1038/srep23451

**Published:** 2016-03-18

**Authors:** Canjun Liu, Yahui Yang, Wenzhang Li, Jie Li, Yaomin Li, Qiyuan Chen

**Affiliations:** 1 School of Chemistry and Chemical Engineering, Central South University, Changsha 410083, China; 2 College of Resources and Environment, Hunan Agricultural University, Changsha 410128, China; 3 Department of Chemistry, University College London, 20 Gordon Street, London, WC1H 0AJ, UK

## Abstract

In this study, Bi_2_S_3_ sensitive layer has been grown on the surface of WO_3_ nanoplate arrays via an *in situ* approach. The characterization of samples were carried out using scanning electron microscopy (SEM), transmission electron microscopy (TEM), X-ray diffraction (XRD) and ultraviolet–visible absorption spectroscopy (UV-vis). The results show that the Bi_2_S_3_ layer is uniformly formed on the surface of WO_3_ nanoplates and less interfacial defects were observed in the interface between the Bi_2_S_3_ and WO_3_. More importantly, the Bi_2_S_3_/WO_3_ films as photoanodes for photoelectrochemical (PEC) cells display the enhanced PEC performance compared with the Bi_2_S_3_/WO_3_ films prepared by a sequential ionic layer adsorption reaction (SILAR) method. In order to understand the reason for the enhanced PEC properties, the electron transport properties of the photoelectrodes were studied by using the transient photocurrent spectroscopy and intensity modulated photocurrent spectroscopy (IMPS). The Bi_2_S_3_/WO_3_ films prepared via an *in situ* approach have a greater transient time constant and higher electron transit rate. This is most likely due to less interfacial defects for the Bi_2_S_3_/WO_3_ films prepared via an *in situ* approach, resulting in a lower resistance and faster carrier transport in the interface between WO_3_ and Bi_2_S_3_.

The limited supply of energy and global climate change caused by burning fossil fuels are two serious challenges faced by humans in the future. Photoelectrochemical (PEC) and photocatalytic (PC) water splitting could be two potential approaches to counter these challenges because solar energy is a clean and inexhaustible energy source^1^,^2^. The PEC water splitting for hydrogen production has attracted extensive attention^3^,^4^, since the first report by Fujishima and Honda in 1972 using TiO_2_ semiconductor material as photoanode^5^. Although many semiconductor materials, such as TiO_2_^6^,^7^, WO_3_^8^,^9^, ZnO^10^,^11^, Fe_2_O_3_^12^,^13^,^14^, CdS^15^,^16^, BiVO_4_^17^,^18^ and so on, can be used as photoelectrode and show PEC activity, most of them have limited utility because of the high charge carrier recombination and large band gap, leading to the low efficiency for PEC water splitting. When a large band gap semiconductor with great surface area is coupled with a small band gap semiconductor with a more negative conduction band (CB) level, the photogenerated electrons in the CB can migrate from the small band gap semiconductor into the large band gap semiconductor and the photogenerated holes in the valence band (VB) move in the opposite direction^19^. Such the heterojunction structure can not only combine with the advantage of two semiconductors but also improve the separation and transport of photogenerated charges^19^,^20^,^21^. The heterojunction films, such as CdS/TiO_2_^22^,^23^,^24^,^25^,^26^, Bi_2_S_3_/TiO_2_^27^,^28^, CdS/ZnO^29^,^30^,^31^ and CdS(CdSe)/ZnO^15^,^32^,^33^ show highly efficient PEC hydrogen generation in the electrolyte containing sulphur ion, due to the expanded the spectral response and efficient carrier separation and transport in the heterojunction.

As a well-known photocatalyst, tungsten trioxide (WO_3_) with a large band gap (2.6 ~ 3.0 eV) has been extensively investigated, because of its excellent photocatalytic activity, high electron mobility and nontoxic nature^8^,^34^,^35^. More recently, the two-dimensional (2D) WO_3_ platelike films show an excellent PEC performance and have attracted intensive attention^36^,^37^,^38^,^39^. Compared to the film comprised of nanoparticles, the 2D platelike films can offer a direct electrical pathway for charge transport, resulting in an enhanced conductivity. It can effectively suppress the recombination of photogenerated electrons and holes in the transfer process^40^,^41^,^42^,^43^.

Bismuth trisulfide (Bi_2_S_3_) is a small band gap semiconductor (~1.3 eV) and has attracted great interest as a sensitizer for PEC and photovoltaic cells recently due to its large absorption coefficient and reasonable IPCE^27^,^44^,^45^,^46^. Moreover, the CB of Bi_2_S_3_ is more negative than that of WO_3_^47^,^48^. It is reasonable to construct the Bi_2_S_3_/WO_3_ heterojunction photoelectrode. Recently, we have reported on the synthesis of Bi_2_S_3_/WO_3_ photoelectrode by a sequential ionic layer adsorption reaction (SILAR) process and the photoelectrodes exhibit an excellent PEC activity^49^. However, it may be weak to the interfacial contact of two components in the heterojunction film prepared by using the SILAR method. Because the SILAR method does not contain calcination process but only the wet-chemical deposition process. The weak interfacial contact will lead to the high resistance in the heterojunction interface. The *in-situ* growth method is considered as a good way to prepare ideal heterojunction with perfect interfacial contact^50^,^51^,^52^. In our study, we found that Bi_2_WO_6_ synthetized from WO_3_ can be used as interim product and Bi_2_S_3_ can be obtained from Bi_2_WO_6_ by a hydrothermal process. Thus, it is feasible to the formation of Bi_2_S_3_/WO_3_ heterojunction by *in-situ* growth method.

Herein, we have rationally designed and developed an *in-situ* growth method using Bi_2_WO_6_ as the interim product to synthesize Bi_2_S_3_ sensitized WO_3_ nanoplate arrays film. The as-prepared films as photoelectrodes show higher PEC activity than that of the Bi_2_S_3_/WO_3_ prepared by SILAR method. This may be because the heterojunction interface with less interfacial defects can be form by *in-situ* growth method, leading to a lower resistance and higher electron transit rate in the heterojunction interface.

## Experiment Section

### Preparation of WO_3_ platelike films

All chemicals were analytical grade. WO_3_ platelike films were prepared by hydrothermal method according to our previous work^39^. In a typical experiment, 0.231 g of sodium tungsten dehydrate (Na_2_WO_4·_2H_2_O) dissolved in 30 mL of deionized water at room temperature. Then, 10 mL of 3 M HCl was added to the solution under constant stirring, followed by the addition of 0.2 g of ammonium oxalate ((NH_4_)_2_C_2_O_4_). After several minutes of stirring, 30 mL of deionized water was added into it with continual stirring for 0.5 h. The as-prepared precursor was transferred into a 100 mL of Teflon-lined stainless autoclave. The FTO substrates with the conducting side facing down were immersed and leaned against the wall of the Teflon-vessel. The hydrothermal synthesis was carried out at 140 °C for 3 h. The as-prepared films were calcined at 500 °C for 1 h.

### Preparation of Bi_2_WO_6_/WO_3_ film

The Bi_2_WO_6_/WO_3_ films were prepared through a simple soaking process. The prepared WO_3_ films were soaked in 0.2 M glacial acetic acid solution of Bi(NO_3_)_3_·5H_2_O for 12 h. To ensure the same thickness Bi_2_WO_6_ layer for all samples, the WO_3_ film was pulled from the solution with a pulling rate of 3 mm/s, not rinsing. Next, the films were dried in 25 °C for 1 h and then calcined at 520 °C in air for 4 h.

### Preparation of Bi_2_S_3_/WO_3_ film

The Bi_2_S_3_/WO_3_ films could be obtained from hydrothermal Bi_2_WO_6_/WO_3_ film. 0.1 g thiourea as the sulphur source was dissolved in 50 mL of DI water at room temperature. Then, 100 μL of 2 M HCl was added to the solution under constant stirring. Next, the solution was transferred into an 80 mL of Teflon-lined stainless autoclave. The Bi_2_WO_6_/WO_3_ film was placed into the autoclave. The hydrothermal synthesis was carried out at 140 °C for 4 h. The as-prepared films were dried at 200 °C for 2 h. For comparison, we also prepared another kind of Bi_2_S_3_/WO_3_ and Bi_2_S_3_ films by the SILAR method according to our previous work^49^. Bi_2_S_3_/WO_3_ films prepared by the SILAR method have been annealed at low-temperature (160 °C, 2 h) after the Bi_2_S_3_ nanoparticles deposition.

### Characterization and photoelectrochemical measurements

The crystalline phase of the sample was characterized by X-ray powder diffraction (XRD, Rigaku D/Max2500, Japan). The UV-Vis diffuse reflection spectra of WO_3_ and Bi_2_WO_6_/WO_3_ film and the Vis-NIR diffuse reflection spectra of Bi_2_S_3_/WO_3_ film were obtained using the UV-Vis 2450 (Shimadzu) and Vis-NIR U-4100 (Hitachi) spectrophotometer, respectively. The diffuse reflection spectra could convert into the absorption spectra by Kubelka-Munk function. Scanning electron microscope (SEM, Nova NanoSEM 230) was used to observe the surface morphology of samples. Transmission electron microscopy (TEM, TECNAI G2 F20, FEI) was operated at an accelerating voltage 200 kV to investigate the microstructure and the crystallinity of samples. On account of the nonuniformity of Bi_2_S_3_ thickness in s-Bi_2_S_3_/WO_3_ film (Supporting Information Fig. S1), the amount of Bi_2_S_3_ in the films was evaluated by the inductively coupled plasma massspectrometry (ICP-MS). The samples with 2 cm^2^ were soaked in 5 ml of 1 M HNO_3_ solution for 12 h and the Bi_2_S_3_ would be dissolved completely. The amount of Bi_2_S_3_ in the film was obtained to characterize the concentration of Bi^3+^ using ICP-MS. The concentration for Bi_2_S_3_/WO_3_ films prepared via an *in situ* approach and the SILAR method is 12.26 and 10.78 mg L^−1^, respectively. The PEC properties of samples were investigated in a typical three-electrode electrochemical cell using an electrochemical analyzer (Zennium, Zahner, Germany). The synthesized films were employed as the working electrode and a platinum foil and an Ag/AgCl/satd. KCl electrode were employed as the counter and reference electrodes, respectively. All electrochemical tests were conducted in an aqueous solution containing 0.1 M Na_2_S and 0.1 M Na_2_SO_3_ (pH ≈ 9). The illumination source was a 150 W xenon lamp (CHF-XM35, Beijing Trusttech Co. Ltd) with a 400 nm cutoff filter to remove UV irradiation. IPCE measurements were carried out using a xenon lamp (150 W, Oriel) with an AM 1.5 filter and a monochromator with a bandwidth of 5 nm. Intensity modulated photocurrent spectroscopy (IMPS) were recorded by using a Zahner CIMPS-2 system. A white light lamp emitting diode (λ = 540 nm) driven by a PP210 was used as lamp and the light intensity is adjusted to 50 mW cm^−2^. Light intensity modulation was driven by current modulation with a depth of 10%. The bias potential is at −0.4 V vs. Ag/AgCl during the IMPS measurement.

## Results and Discussion

**Figure 1:**
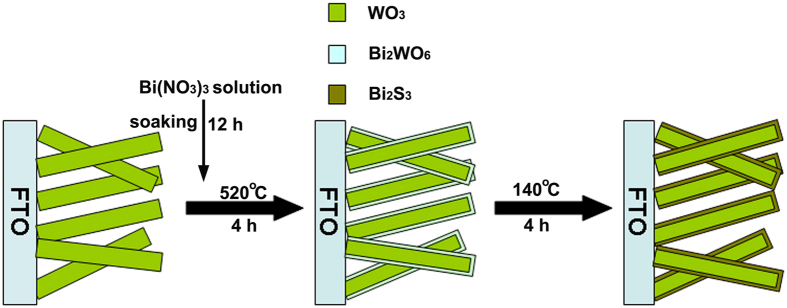
Depiction of the synthesis process of Bi_2_S_3_/WO_3_ films.

**Figure 2:**
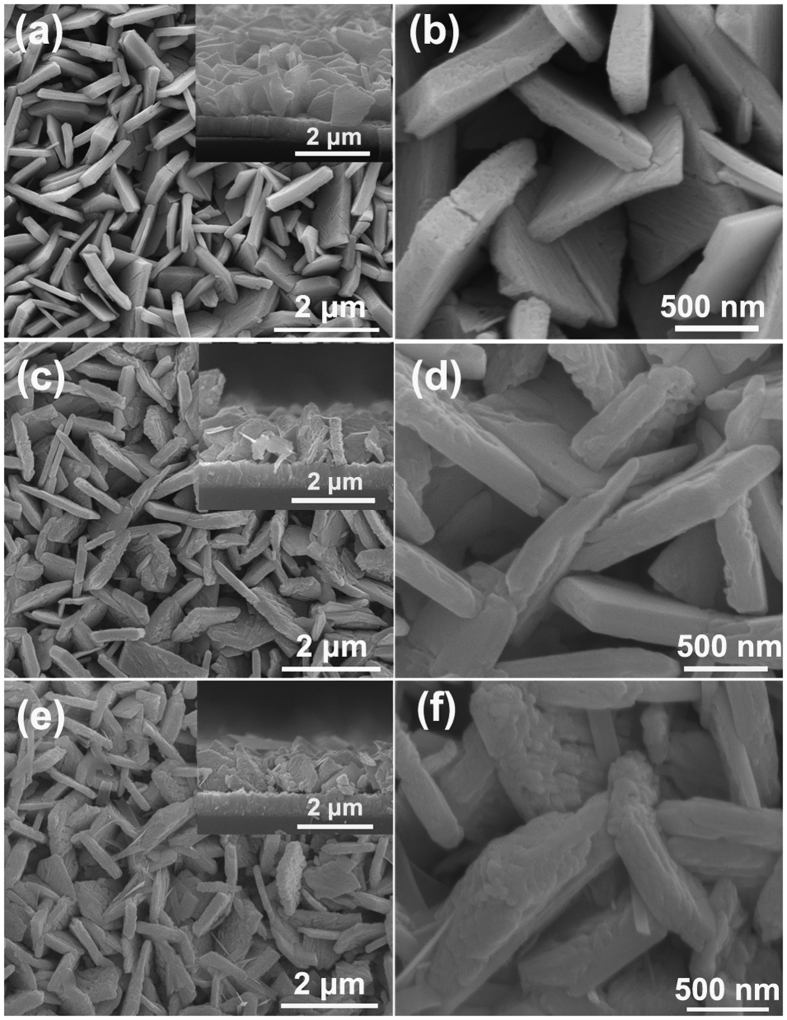
SEM images of (**a**,**b**) the WO_3_, (**c**,**d**) Bi_2_WO_6_/WO_3_ and (**e**,**f**) Bi_2_S_3_/WO_3_ film.

The depiction of the fabrication process for Bi_2_S_3_/WO_3_ films by *in-situ* growth method and surface morphology change of sample for each step are shown in Figs 1 and 2, respectively. First, the platelike WO_3_ arrays films were obtained via a hydrothermal process. The SEM images of platelike WO_3_ film in Fig. 2a,b reveal that the highly dense and uniform vertical nanoplates with edge length of 0.5–1.5 μm and thickness of 50–200 nm grown on a FTO substrate. Second, the Bi_2_WO_6_ was formed on WO_3_ nanoplate by a simple soaking and calcined process. The SEM images of Bi_2_WO_6_/WO_3_ film shown in Fig. 2c,d. As shown in Fig. 2a–d, the surface morphology of Bi_2_WO_6_/WO_3_ is different from that of the pristine WO_3_. Compared with the pristine WO_3_, the surface of Bi_2_WO_6_/WO_3_ nanoplates became rough and the surface textures reduce obviously. It may because the formation of the bigger molecule Bi_2_WO_6_ lead to the surface swell. Third, Bi_2_S_3_/WO_3_ films can be obtained from hydrothermal Bi_2_WO_6_/WO_3_ film. During the hydrothermal process, the S^2−^ can be generated by the decomposition of thiourea, and then the Bi_2_WO_6_ will react with the S^2−^ to form the Bi_2_S_3_ on the surface of WO_3_ nanoplates^53^. It can be seen from Fig. 2e,f that the surface of Bi_2_S_3_/WO_3_ nanoplates is rougher than that of Bi_2_WO_6_/WO_3_.

**Figure 3:**
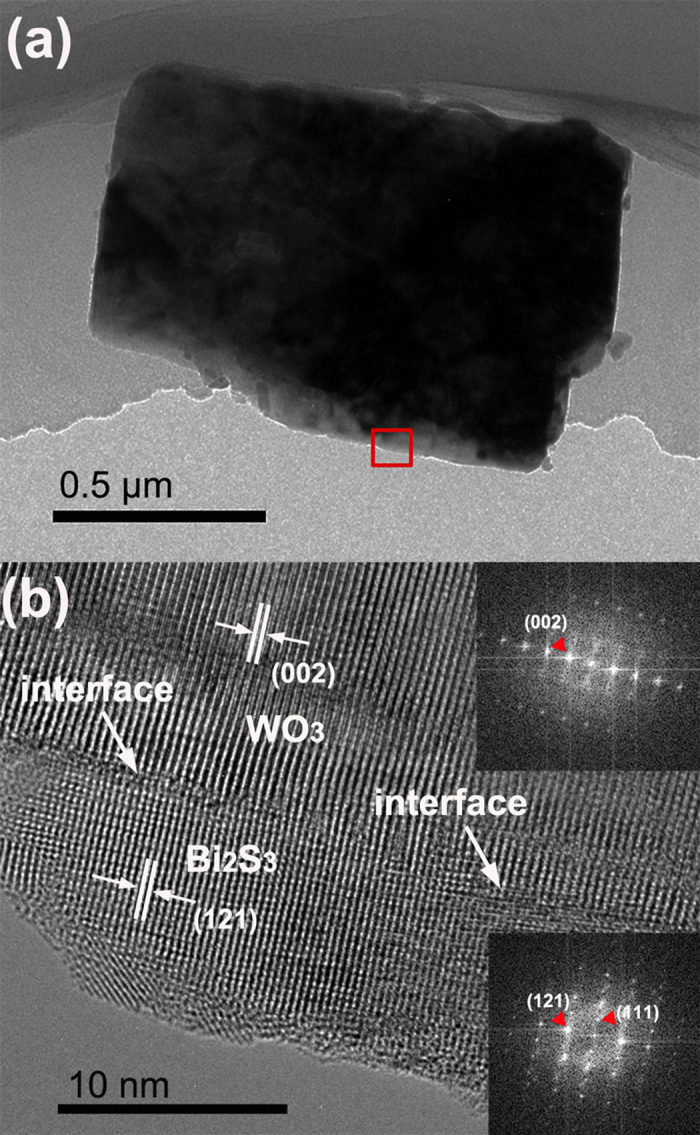
(**a**) TEM image of a Bi_2_S_3_/WO_3_ plate, (**b**) HRTEM image of the interface between Bi_2_S_3_ and WO_3_.

**Figure 4:**
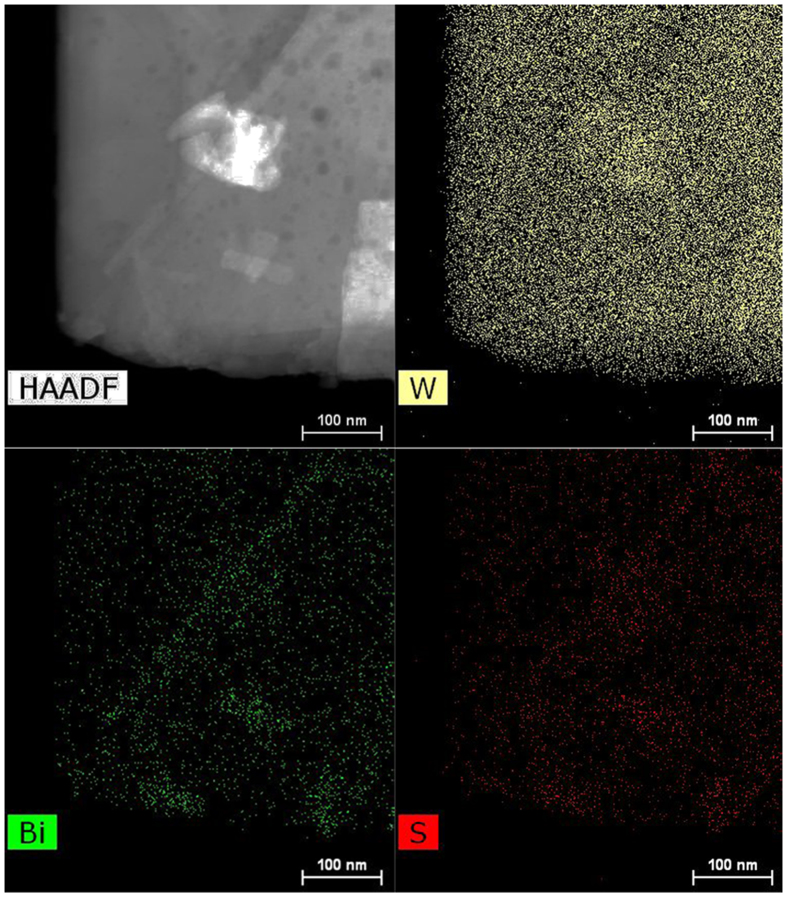
(**a**) Scanning transmission electron microscopy (STEM) image of Bi_2_S_3_/WO_3_ plate and (**b**–**d**) the corresponding elemental mapping images.

Further, the transmission electron microscopy (TEM) was used to identify the elaborate structure of the composite nanoplates. Typical WO_3_ nanoplate is clearly shown in the low-resolution TEM image (Fig. S2). The uniform lattice fringe can be observed over an entire primary nanoplate from the high resolution TEM (HR-TEM) image of WO_3_ (Fig. S2b), revealing a single crystalline characteristic. The distance between each fringe is about 0.308 nm, matching well with the (112) of monocline phase WO_3_ (JCPDS 83-0950). The HR-TEM image recorded on the rim of a Bi_2_WO_6_/WO_3_ nanoplate was shown in Fig. S2c. The lattice spacing of 0.375 nm is consistent with the interplanar spacings of (111) planes of orthorhombic Bi_2_WO_6_. In addition, the lattice fringes with the spacing of 0.384 and 0.335 nm are in good agreement with the (002) and (120) planes of WO_3_, respectively. The TEM results for the Bi_2_WO_6_/WO_3_ nanoplate can confirm that Bi_2_WO_6_ layer can be formed on the surface of WO_3_ plate via an *in situ* approach. The HR-TEM image of the interface between Bi_2_S_3_ and WO_3_ is shown in Fig. 3b. The exact interface between the two phases is clearly shown in the image. The lattice spacing observed to be 0.384 corresponds to (002) plane of monocline phase WO_3_ (JCPDS 83–0950) and the lattice spacing of 0.312 nm is assigned to the (121) plane of Bi_2_S_3_ (JCPDS No. 84–0279). The two corresponding fast Fourier transform patterns (inset in Fig. 3b) confirm the single-crystal structure of the WO_3_ nanoplate and Bi_2_S_3_ layer. Especially, misfit dislocations or defect region are not seen near the physical interface, indicating the formation of an interface with less interfacial defects between Bi_2_S_3_ and WO_3_. Spatial elemental mapping was also performed on a single Bi_2_S_3_/WO_3_ plate to reveal the distribution of the two phases in the heterostructure. The mapping results confirm that the Bi_2_S_3_ is uniformly coated on WO_3_ plate to form core/shell structure (Fig. 4a–d).

**Figure 5:**
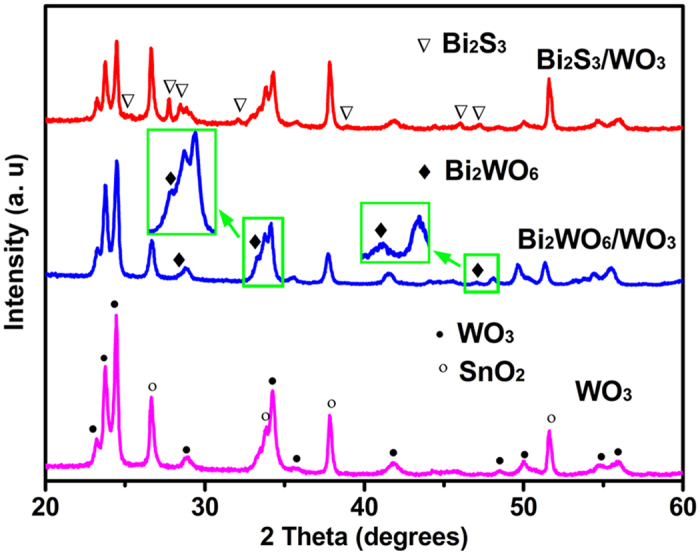
XRD patterns the WO_3_, Bi_2_WO_6_/WO_3_ and Bi_2_S_3_/WO_3_ film.

**Figure 6:**
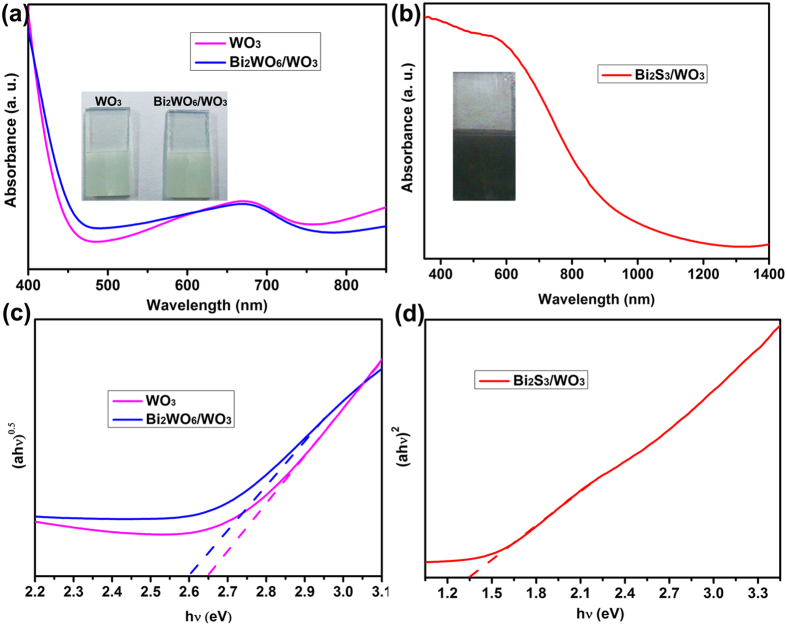
(**a**,**b**) UV−vis and Vis-NIR absorption spectra of the WO_3_, Bi_2_WO_6_/WO_3_ and Bi_2_S_3_/WO_3_ films. (**c**,**d**) Tauc plots of the WO_3_, Bi_2_WO_6_/WO_3_ and Bi_2_S_3_/WO_3_ films.

The XRD was employed to examine the crystal structures and phase purity of the sample films. The XRD patterns of pristine WO_3_, Bi_2_WO_6_/WO_3_ and Bi_2_S_3_/WO_3_ films are shown in Fig. 5a. The peaks of pristine WO_3_ can be indexed to the diffractions from the monocline phase WO_3_ (JCPDS 83–0950). The three small diffraction peaks of the Bi_2_WO_6_/WO_3_ sample can be observed at 28.38, 33.05 and 47.16^o^, attributed to the characteristic diffraction peak of Bi_2_WO_6_ (JCPDS No. 73–2020). In addition, many weak diffraction peaks observed from the Bi_2_S_3_/WO_3_ sample, are identical to Bi_2_S_3_ (JCPDS No. 84–0279). The result indicates the formation of Bi_2_S_3_ on the surface of WO_3_ plate. The optical behavior of the prepared films was evaluated by using UV-vis and Vis-NIR absorption spectroscopy. Figure 6 shows the absorption spectra and Tauc plots of the WO_3_, Bi_2_WO_6_/WO_3_ and Bi_2_S_3_/WO_3_ film. For the pristine WO_3_, a clear absorption edge about 460 nm can be observed, corresponding to its indirect band gap energy (Fig. 6a). There is a weak red shift in the absorption spectra after the *in situ* growth of Bi_2_WO_6_ on the surface of WO_3_ plates. Differently, the Bi_2_S_3_/WO_3_ film shows strong absorption intensity at wavelength of ~950 nm (Fig. 6b), which is consistent with the reported absorption spectra of the Bi_2_S_3_/WO_3_ film^49^. The photograph of sample also supports the above results. As shown in Fig. 6 (inset), the color of the Bi_2_WO_6_/WO_3_ film almost is the same with the pristine WO_3_ film (Fig. 6a inset), while the Bi_2_S_3_/WO_3_ film shows black (Fig. 6b inset). In addition, the optical bandgap of samples have been calculated by the Tauc equation. As shown in the Fig. 6c,d, the bandgap energy of WO_3_, Bi_2_WO_6_/WO_3_ and Bi_2_S_3_/WO_3_ film is 2.60, 2.65 and 1.35 eV, respectively.

**Figure 7:**
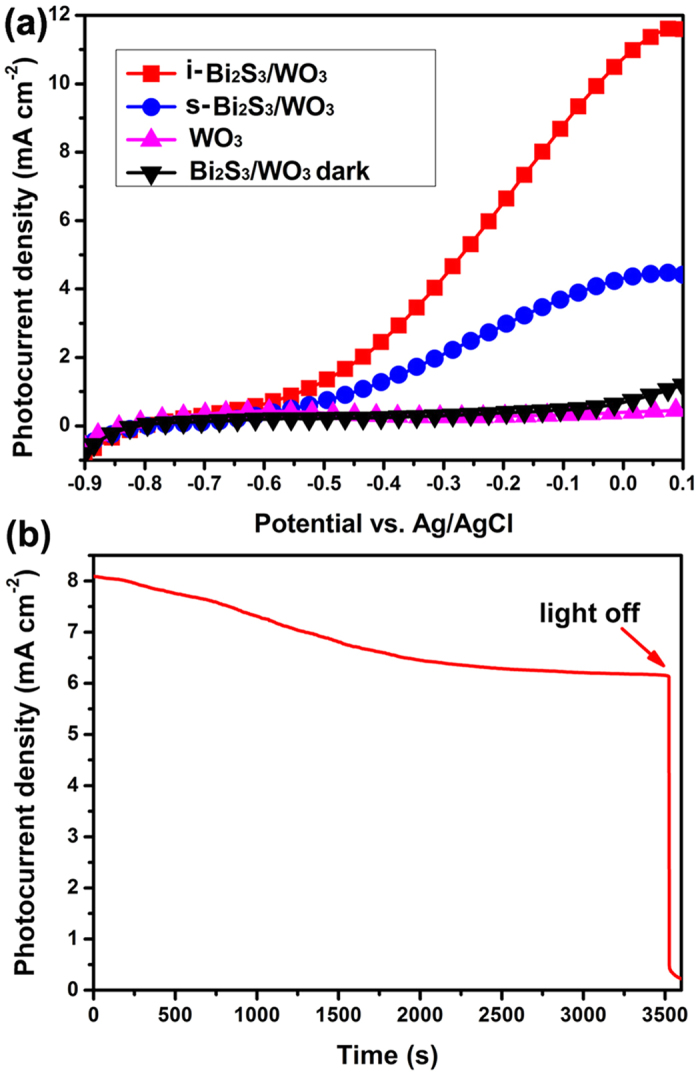
(**a**) LSV scans of s-Bi_2_S_3_/WO_3_ and i-Bi_2_S_3_/WO_3_ films and (**b**) the photocurrent−time plot of i-Bi_2_S_3_/WO_3_ photoelectrodes.

The PEC measurements were implemented in a three-electrode PEC cell using the prepared films as photoanodes under illumination of visible light. Linear sweep voltammetry (LSV) was employed to evaluate the PEC performance of films. For comparison, the Bi_2_S_3_/WO_3_ films prepared by the SILAR method also were used as photoanodes and denoted as s-Bi_2_S_3_/WO_3_ photoelectrodes. Likewise, the Bi_2_S_3_/WO_3_ prepared films by *in-situ* growth method were denoted as i-Bi_2_S_3_/WO_3_ photoelectrodes. Their PEC performances were recorded in the same condition. The LSV of photoelectrodes obtained in the dark and under illumination is shown in Fig. 7a. Under light illumination, the photocurrent density of the pristine WO_3_ photoelectrodes is negligible, while the photoelectrodes sensitized by Bi_2_S_3_ show significant photocurrent generation. This is because of the poor visible-light response for WO_3_. Compared to the s-Bi_2_S_3_/WO_3_ photoelectrodes (3.9 mA cm^−2^ at −0.1 V vs Ag/AgCl), the i-Bi_2_S_3_/WO_3_ photoelectrodes show a higher photocurrent density (8.0 mA cm^−2^ at −0.1 V vs Ag/AgCl). In addition, the stability of i-Bi_2_S_3_/WO_3_ photoelectrode is tested by performing long-duration PEC experiments. The testing experiment is carried out at the −0.1 V vs Ag/AgCl under continuous illumination and lots of H_2_ bubbles can be found on the surface of Pt electrode throughout the entire test. The result is shown in Fig. 7b. The photocurrent decreased by 23% after a 3600 s operation, and could remain stable in the latter time period.

To investigate the quantitative correlation between the wavelength of the incident light and the PEC activity, incident photon-to-current conversion efficiency (IPCE) measurement was performed at a bias of −0.5 V vs. Ag/AgCl. The IPCE were estimated by the following relation^15^,^17^:


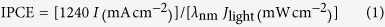


**Figure 8:**
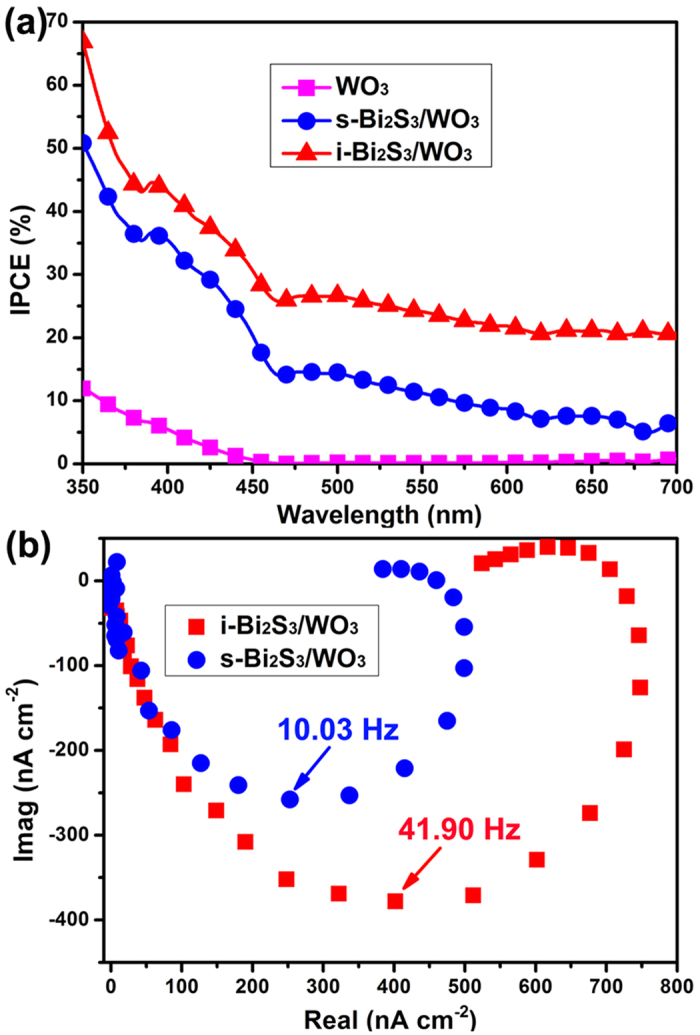
(**a**) IPCE spectra and (**b**) complex plane plot of the IMPS response for s-Bi_2_S_3_/WO_3_ and i-Bi_2_S_3_/WO_3_ photoelectrodes.

Where *I* is the photocurrent density, *λ* is the incident light wavelength, and *J*_*light*_ is the incident light power density. The IPCE of the WO_3_, s-Bi_2_S_3_/WO_3_ and i-Bi_2_S_3_/WO_3_ photoelectrodes are shown in Fig. 8a. The pristine WO_3_ photoelectrode exhibits the photoresponse only at the wavelength range of ~460 nm, because of the large band gap of WO_3_ (≥2.6 eV). Relative to the pristine WO_3_ photoelectrode, the photoelectrode decorated by Bi_2_S_3_ shows enhanced IPCE in the entire testing wavelength region due to the increased absorption by the Bi_2_S_3_. Importantly, the i-Bi_2_S_3_/WO_3_ photoelectrode shows the highest IPCE value, which is in accordance with the LSV result.

In order to understand the reason that the i-Bi_2_S_3_/WO_3_ photoelectrode has an enhanced PEC property, the electron transport properties of the photoelectrodes were investigated by using intensity modulated photocurrent spectroscopy (IMPS) analysis. Figure 8b shows the complex plane plot of the IMPS response for s-Bi_2_S_3_/WO_3_ and i-Bi_2_S_3_/WO_3_ photoelectrodes at −0.4 V vs. Ag/AgCl. The IMPS is popularly used to characterize the electron transport of PEC cells^54^,^55^. The electron transport time (*τ*_d_) is the average time photogenerated electrons need to reach the back contact and can be obtained from the IMPS result by the following formula^19^,^28^,^56^,^57^:


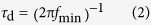


where *f*_min_ is the frequency at the imaginary minimum. *τ*_d_ of the s-Bi_2_S_3_/WO_3_ and i-Bi_2_S_3_/WO_3_ photoelectrode are 15.87 and 3.80 ms, respectively. The results suggest the electron transit rate in i-Bi_2_S_3_/WO_3_ photoelectrode is faster than in the s-Bi_2_S_3_/WO_3_ photoelectrode. It may be attributed to the stronger contact and lower resistance in the interface between Bi_2_S_3_ and WO_3_ for the i-Bi_2_S_3_/WO_3_ photoelectrode. To further verify the IMPS results, we also measured IMPS at −0.6 and −0.2 V vs. Ag/AgCl and the results are shown in Fig. S3. Similarly, the *τ*_d_ of i-Bi_2_S_3_/WO_3_ photoelectrodes are smaller than that of s-Bi_2_S_3_/WO_3_ photoelectrodes at the same potential due to the higher *f*_min_.

To further support the above ideas, the transient photocurrent plots were measured at −0.7 V and −0.5 V vs. Ag/AgCl and were shown in Fig. 8. The average transient time constant (*τ*_t_) of photoelectrodes can be calculated and obtained from the transient photocurrent plots by using the kinetic equations as follows^58^,^59^,^60^,^61^:


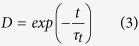


Where *D* is defined as


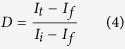


**Figure 9:**
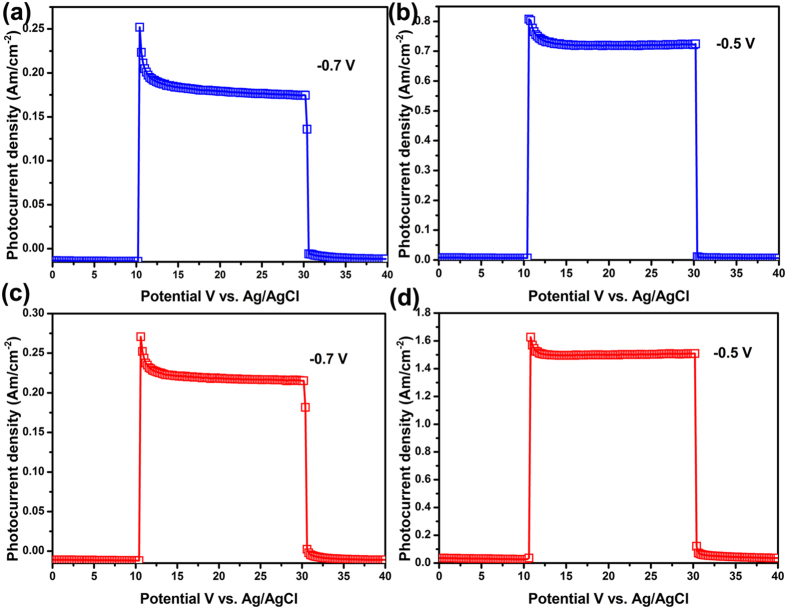
The transient photocurrent plots of (**a**,**b**) s-Bi_2_S_3_/WO_3_ and (**c**,**d**) i-Bi_2_S_3_/WO_3_ photoelectrodes at −0.7 and −0.5 V vs. Ag/AgCl, respectively.

Where *I* is the current, i and *f* are related to the initial and final steady states, and *t* denotes time, respectively. The transient time constant, *τ*_*t*_, can be defined as the time at ln D = −1. Generally, a longer transient time constant implies a smaller extent of recombination and the transient time may be treated as a lifetime of the photogenerated carriers. As shown in Fig. 9, the photocurrents were approximately zero in the dark. Once illumination, the photocurrents rapidly increase, and then begin to decay. The decay of the photocurrents implies that the recombination of the photogenerated carriers occurs. The *τ*_*t*_ values of the s-Bi_2_S_3_/WO_3_ and i-Bi_2_S_3_/WO_3_ photoelectrode are shown in Table 1. The i-Bi_2_S_3_/WO_3_ photoelectrode has longer the transient time than that of the s-Bi_2_S_3_/WO_3_ photoelectrode, suggesting a smaller extent of recombination for the i-Bi_2_S_3_/WO_3_ photoelectrode. This may be because of the faster carries transit rate for the i-Bi_2_S_3_/WO_3_ photoelectrode, resulting in the reducing of the recombination of the photogenerated carriers.

**Figure 10:**
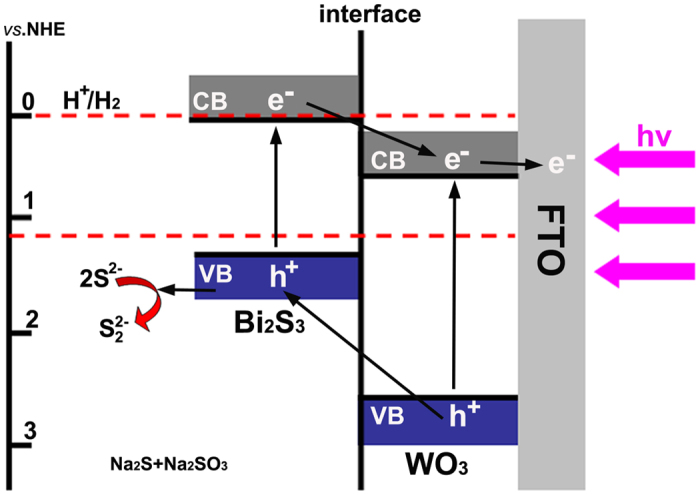
The schematic illustration of the charge transfer of the photoelectrode.

To better understand the charge separation and transfer process in the PEC cells, the schematic illustration of the charge transfer of the photoelectrode is illustrated in Fig. 10. Under visible light irradiation, the electrons are excited from the valence band (VB) of WO_3_ and Bi_2_S_3_ to their conduction band (CB). The accumulated electrons at the CB of Bi_2_S_3_ easily move to the CB of WO_3_ due to the more negative CB of Bi_2_S_3_, and then are collected by the FTO conductor^49^. It can also be supported from the analysis of Mott−Schottky results (Supporting Information Fig. S4). At the same time, the holes formed in the valence band (VB) of WO_3_ and Bi_2_S_3_ will be transferred to the semiconductor/electrolyte interface in the opposite direction and react with S^2−^ to avoid the photocorrosion of Bi_2_S_3_. The electrons in the FTO are migrated to the Pt electrode/electrolyte interface by the external bias voltage and will reduce the H_2_O to H_2_. It should be noted that the PEC cells discussed here need the presence of Na_2_SO_3_ and Na_2_S as the sacrificial reductants to steadily generate H_2_, which has been widely reported^15^,^24^,^31^,^33^,^62^. The addition of the reductant will not affect the comparison of the PEC performance of photoelectrodes, but the development of efficient PEC cell with no need of sacrificial reductants is important and urgent for PEC water splitting, and yet still very challenging.

## Conclusions

In conclusion, we have synthesized Bi_2_S_3_ sensitized WO_3_ nanoplate arrays films via an *in situ* approach. The film characterization results suggest that the Bi_2_S_3_ layer was uniformly formed on the surface of WO_3_ nanoplates. The prepared films were used as photoanodes and the PEC performance was studied. The Bi_2_S_3_/WO_3_ films prepared via an *in situ* approach have a higher photocurrent density (8.0 mA cm^−2^ at −0.1 V vs Ag/AgCl) than that of the Bi_2_S_3_/WO_3_ films prepared by SILAR method. This may be due to the stronger contact interface for the Bi_2_S_3_/WO_3_ films prepared via an *in situ* approach, resulting in a higher electron transit rate and the reduced photogenerated carrier recombination. This versatile preparation method has potential to be applied in the synthesis of other hybrid films with less interfacial defects.

## Additional Information

**How to cite this article**: Liu, C. *et al*. In situ synthesis of Bi_2_S_3_ sensitized WO_3_ nanoplate arrays with less interfacial defects and enhanced photoelectrochemical performance. *Sci. Rep.* **6**, 23451; doi: 10.1038/srep23451 (2016).

## Supplementary Material

Supplementary Information

## Figures and Tables

**Table 1:** The average transient time constant of s-Bi_2_S_3_/WO_3_ (*τ*_s_) and i-Bi_2_S_3_/WO_3_ (*τ*_i_) photoelectrodes.

**Potential**	**−0.7 V**	**−0.5 V**
τ_s_ (s)	10.4	11.2
τ_i_ (s)	12.1	12.7
