# Supplementary material for: In situ synthesis of Bi2S3 sensitized WO3 nanoplate arrays with less interfacial defects and enhanced photoelectrochemical performance
Source: Sci Rep. 2016 Mar 18;6:23451. doi: 10.1038/srep23451 (PMC4796909; doi:10.1038/srep23451)
Supplement: Supplementary Information [file srep23451-s1.pdf]

In situ synthesis of Bi_2S_3 sensitized WO_3 nanoplate arrays with less interfacial defects and enhanced photoelectrochemical performance

Canjun Liu [1], Yahui Yang [2], Wenzhang Li [1,*], Jie Li [1,*], Yaomin Li [3], Qiyuan Chen [1]

[1] School of Chemistry and Chemical Engineering, Central South University, Changsha 410083 China

[2] College of Resources and Environment, Hunan Agricultural University, Changsha 410128, China

[3] Department of Chemistry, University College London, 20 Gordon Street, London, WC1H 0AJ, UK

[*]Corresponding author. Tel.: +86 731 8887 9616; fax: +86 731 8887 9616.

Correspondence and requests for materials should be addressed to W.L (liwenzhang@csu.edu.cn) or J.L (lijieliu@csu.edu.cn)

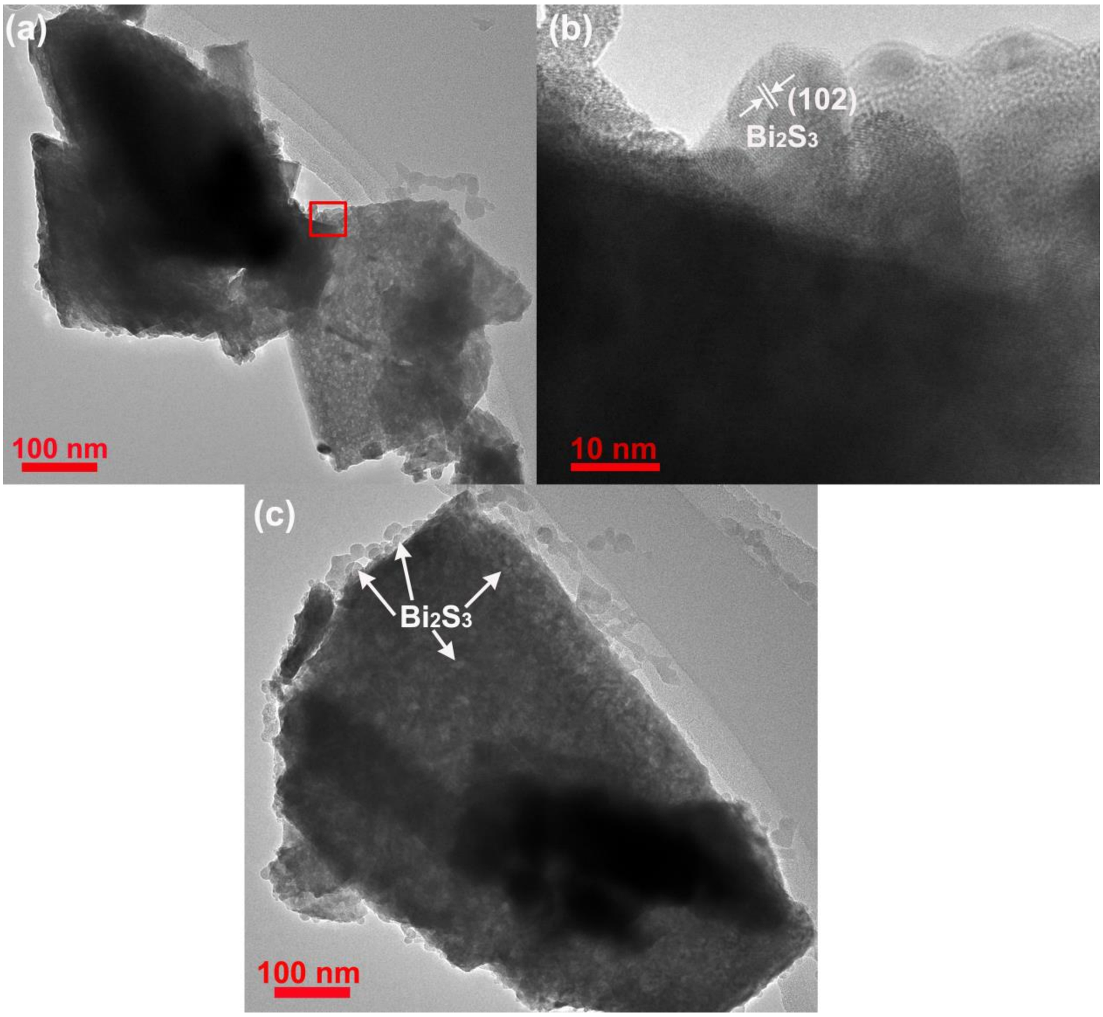

Figure S1 TEM and HR-TEM image of s-Bi_2S_3/WO_3 plates

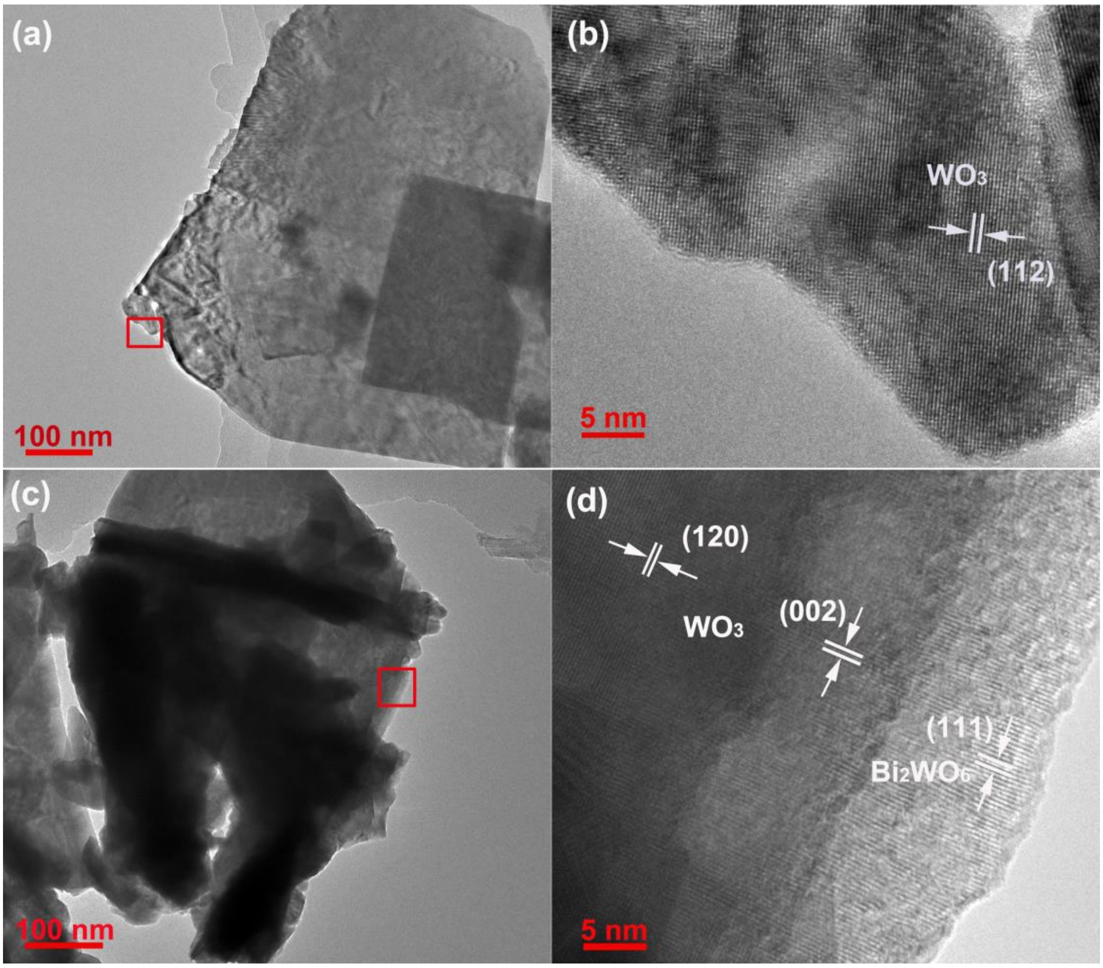

Figure S2 TEM and HR-TEM image of (a), (b) WO_3 and (c), (d) Bi_2WO_6/WO_3 plates

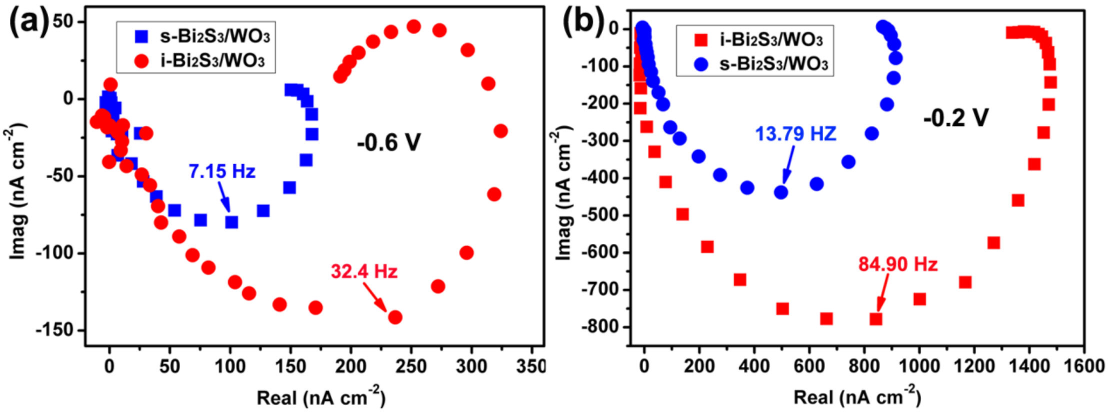

Figure S3 The complex plane plots of the IMPS response at (a) -0.6 and (b) -0.2 V vs. Ag/AgCl, respectively

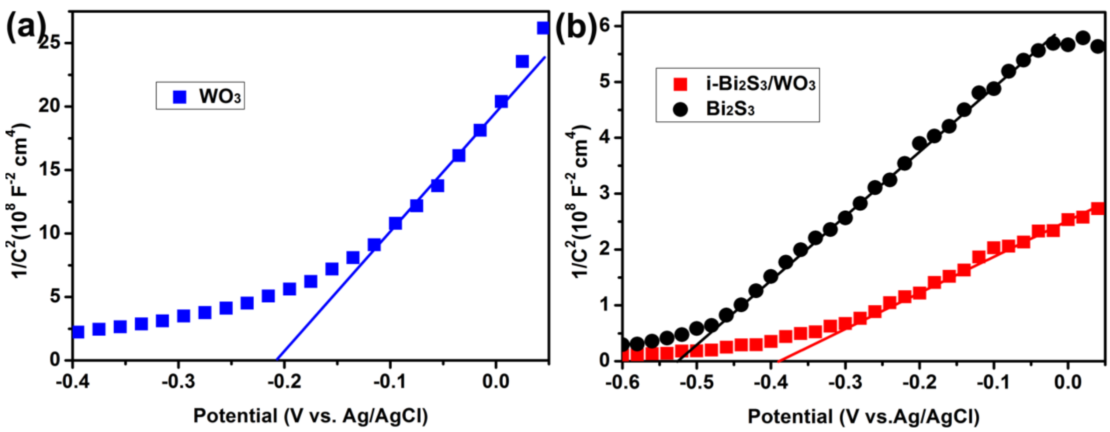

Figure S4 Mott–Schottky plots of the photoelectrodes
